# Lymph node assessment in patients with early-stage breast cancer: the current role of imaging methods

**DOI:** 10.1590/0100-3984.2024.57.e11-en

**Published:** 2024-11-29

**Authors:** Sandra Regina Campos Teixeira, Almir Galvão Vieira Bitencourt

**Affiliations:** 1 Department of Radiology, Hospital Unimed Campinas, Campinas, SP, Brazil; 2 Gynecological and Breast Oncology Division, Department of Obstetrics and Gynecology, Faculdade de Ciências Médicas da Universidade Estadual de Campinas (FCM-Unicamp), Campinas, SP, Brazil; 3 Department of Imaging, A.C.Camargo Cancer Center, São Paulo, SP, Brazil; 4 DASA, São Paulo, SP, Brazil

Axillary lymph node involvement is the main route of dissemination of breast cancer, preceding distant metastases in most cases^(1)^. Traditionally, lymph node staging has been one of the main parameters used in order to define the prognosis and treatment of patients with breast cancer. However, surgical management of the axillae has changed significantly in recent years, with a growing trend towards de-escalation of surgical treatment, especially in patients with early-stage tumors and clinically negative axillae^(2)^. After randomized controlled trials such as the ACOSOG Z0011 trial^(3)^, the NSABP B-32 trial^(4)^, and, most recently, the SOUND trial^(5)^, many patients who previously required axillary dissection began to undergo only sentinel lymph node examination, or even no surgical intervention in selected cases. Therefore, the role of different imaging methods has become even more important for appropriate staging and therapeutic planning^(5)^.

In a study recently published in Radiologia Brasileira, Batista et al.^(6)^ evaluated the performance of magnetic resonance imaging (MRI) to detect axillary metastases in patients with early-stage (T1 or T2) invasive breast carcinomas and clinically negative axillae. A total of 119 patients who underwent preoperative MRI and a surgical approach to the axillae were evaluated, of whom 20 (16.5%) had histologically confirmed axillary metastases. The results demonstrated that MRI has low sensitivity (35.0%), especially for the diagnosis of micrometastases, although it was found to have high specificity (81.2%) and a high negative predictive value (86.3%). The authors emphasize that, in this population of patients with early-stage tumors, the lymph node tumor burden is generally lower, with little or no morphological alteration, which impedes evaluation by imaging.

Mammography (with or without contrast) has major limitations in axillary evaluation, showing only part of axillary level I in the mediolateral oblique view, often requiring additional views, such as an axillary view. Multiplanar methods such as MRI, computed tomography (CT), positron emission tomography/ CT (PET/CT), and PET/MRI have the advantage of three-dimensional evaluation and high anatomical resolution, although they have major limitations, such as their high cost, variable availability, and dependence on socioeconomic conditions. Breast MRI has some limitations in the evaluation of axillary lymph nodes, mainly due to the field of view of the examination, which may not fully include the axillae or can present a low signal-to-noise ratio, impairing the evaluation of lymph nodes in the upper portions (axillary levels II and III). Although chest CT and PET/CT can also be used for lymph node evaluation with good accuracy, they are generally indicated only in patients with advanced disease and have the additional disadvantage of using ionizing radiation^(7-9)^.

The most cost-effective and accurate imaging method for assessing axillary lymph node involvement is ultrasound, which is superior to MRI and PET/CT for that purpose^(10)^. Another major advantage of ultrasound is its ability to guide biopsies, if necessary, which is not possible with other methods because of anatomical characteristics that increase the risk of vascular or pleuropulmonary lesion. However, it is important that the examination be performed by a professional trained for this type of assessment, using a standardized protocol^(11)^. Morphological alterations considered suspicious on ultrasound are cortical thickness > 3 mm, eccentric cortical thickening, displacement or loss of the fatty hilum, globular or irregular shape, and indistinct margins^(8)^. For current staging, it is important that the number of suspicious lymph nodes and the axillary levels affected are also described. Recent studies suggest that patients with early-stage tumors without suspicious axillary lymph nodes on ultrasound may be spared from undergoing sentinel lymph node testing, without reducing disease-free survival^(5)^.

In conclusion, lymph node imaging will be increasingly important in the preoperative staging of patients with earlystage breast cancer. Breast MRI has limited accuracy in this population, and ultrasonography is still the method of choice for identifying suspicious lymph nodes, which has a significant impact on treatment planning.

## References

[1] Bitencourt A, Saccarelli CR, Morris EA (2020). Regional lymph node involvement among patients with de novo
metastatic breast cancer. JAMA Netw Open.

[2] Williams AD, Weiss A. (2024). Recent advances in the upfront surgical management of the axilla
in patients with breast cancer. Clin Breast Cancer.

[3] Giuliano AE, Ballman KV, McCall L (2017). Effect of axillary dissection vs no axillary dissection on
10-year overall survival among women with invasive breast cancer and
sentinel node metastasis: The ACOSOG Z0011 (Alliance) Randomized Clinical
Trial. JAMA.

[4] Krag DN, Anderson SJ, Julian TB (2010). Sentinel-lymph-node resection compared with conventional
axillary-lymph-node dissection in clinically nodenegative patients with
breast cancer: overall survival findings from the NSABP B-32 randomised
phase 3 trial. Lancet Oncol.

[5] Gentilini OD, Botteri E, Sangalli C (2023). Sentinel lymph node biopsy vs no axillary surgery in patients
with small breast cancer and negative results on ultrasonography of axillary
lymph nodes: The SOUND Randomized Clinical Trial. JAMA Oncol.

[6] Batista TC, Lauar MCV, Federicci EEF (2024). Magnetic resonance imaging evaluation of axillary lymph nodes in
patients with early-stage invasive breast cancer. Radiol Bras.

[7] Sun SX, Moseley TW, Kuerer HM (2020). Imaging-based approach to axillary lymph node staging and
sentinel lymph node biopsy in patients with breast cancer. AJR Am J Roentgenol.

[8] Chung HL, Le-Petross HT, Leung JWT. (2021). Imaging updates to breast cancer lymph node
management. Radiographics.

[9] Ecanow JS, Abe H, Newstead GM (2013). Axillary staging of breast cancer: what the radiologist should
know. Radiographics.

[10] Le Boulc’h M, Gilhodes J, Steinmeyer Z (2021). Pretherapeutic imaging for axillary staging in breast cancer: a
systematic review and meta-analysis of ultrasound, MRI and FDG
PET. J Clin Med.

[11] Cocco G, Ricci V, Ricci C (2023). Ultrasound imaging of the axilla. Insights Imaging.

